# Eyes shut homolog (EYS) interacts with matriglycan of O-mannosyl glycans whose deficiency results in EYS mislocalization and degeneration of photoreceptors

**DOI:** 10.1038/s41598-020-64752-4

**Published:** 2020-05-08

**Authors:** Yu Liu, Miao Yu, Xuanze Shang, My Hong Hoai Nguyen, Shanmuganathan Balakrishnan, Rachel Sager, Huaiyu Hu

**Affiliations:** 1 Center for Vision Research, Departments of Neuroscience and Physiology and of Ophthalmology and Visual Sciences, Upstate Medical University, Syracuse, NY 13210 USA; 2 Present Address: Department of Biological Sciences, State University of New York at Plattsburgh, 101 Broad St., Plattsburgh, New York, 12901 USA

**Keywords:** Neuroscience, Cell death in the nervous system

## Abstract

Mutations in eyes shut homolog (EYS), a secreted extracellular matrix protein containing multiple laminin globular (LG) domains, and in protein O-mannose β1, 2-N-acetylglucosaminyl transferase 1 (POMGnT1), an enzyme involved in O-mannosyl glycosylation, cause retinitis pigmentosa (RP), RP25 and RP76, respectively. How EYS and POMGnT1 regulate photoreceptor survival is poorly understood. Since some LG domain-containing proteins function by binding to the matriglycan moiety of O-mannosyl glycans, we hypothesized that EYS interacted with matriglycans as well. To test this hypothesis, we performed EYS Far-Western blotting assay and generated *pomgnt1* mutant zebrafish. The results showed that EYS bound to matriglycans. *Pomgnt1* mutation in zebrafish resulted in a loss of matriglycan, retention of synaptotagmin-1-positive EYS secretory vesicles within the outer nuclear layer, and diminished EYS protein near the connecting cilia. Photoreceptor density in 2-month old *pomgnt1* mutant retina was similar to the wild-type animals but was significantly reduced at 6-months. These results indicate that EYS protein localization to the connecting cilia requires interaction with the matriglycan and that O-mannosyl glycosylation is required for photoreceptor survival in zebrafish. This study identified a novel interaction between EYS and matriglycan demonstrating that RP25 and RP76 are mechanistically linked in that O-mannosyl glycosylation controls targeting of EYS protein.

## Introduction

Retinitis pigmentosa (RP) is, genetically, a very heterogeneous group of blinding diseases resulting from photoreceptor degeneration. It affects roughly 1 in 4,000 people worldwide with no effective therapy, according to the National Eye Institute^1^. Like usherin (USH2A), mutations in the extracellular matrix protein eyes shut homolog (EYS)^2,3^ are a common cause of the autosomal recessive form of the disease^4–10^, retinitis pigmentosa 25 (RP25, OMIM#612424). Some patients with EYS mutations exhibit cone-rod dystrophy^11^. The EYS gene encodes a large secreted protein comprised of two types of structural domains, laminin globular (LG) domains and EGF repeats. The functions of these domains in EYS are unknown. Interestingly, some mammalian clades, including mouse, have lost the EYS locus^2^, limiting our ability to investigate how EYS contributes to photoreceptor survival in the rodent. Thus, the zebrafish, which has an EYS locus, has been used to explore EYS function in the retina^12–14^. EYS-deficient zebrafish exhibits photoreceptor degeneration, confirming that EYS is essential for photoreceptor survival. Previous studies indicate that EYS protein is highly enriched near the connecting cilium/transition zone (CC/TZ) and is required for maintaining the structural integrity of the ciliary pocket^12^. However, the mechanisms that control its specific localization are unclear.

Congenital muscular dystrophies with brain malformation and retinal atrophy such as muscle-eye-brain disease (MEB) are mainly caused by mutations in enzymes involved in O-mannosyl glycosylation (reviewed in^15^). MEB is often caused by null mutations in one of these enzymes called protein O-mannose N-acetylglucosaminyltransferase 1 (POMGnT1)^16^. Retinal response to light stimulation in MEB patients is severely reduced or completely abolished on electroretinograms^17,18^. Hypomorphic POMGnT1 mutations are also associated with several cases of retinitis pigmentosa (RP76, OMIM#617123)^19,20^. These reports suggest that POMGnT1 mutation causes photoreceptor degeneration in humans. However, there is no evidence of photoreceptor degeneration in POMGnT1 knockout mice although they exhibit other retinal abnormalities including a thinner retina, disrupted inner limiting membrane, ribbon synaptic transmission defects, and reactive gliosis^21–23^. Thus, POMGnT1 mutation as a cause of photoreceptor degeneration has not been confirmed in an animal model.

Synthesis of O-mannosyl glycans is initiated by the protein O-mannosyltransferase 1 and 2 (POMT1/2) complex in the ER^24^. POMGnT1 and POMGnT2, in the Golgi apparatus, catalyze the addition of a β1,2- and a β1,4-linked N-acetylglucosamine off O-mannose. The β1,4 branch is subsequently modified by a series of Golgi enzymes that include β1,3-N-acetylgalactosaminyl transferase 2, fukutin, fukutin-related protein, TMEM5, and β1,4-glucoronic acid transferase 2. Two additional Golgi enzymes, like-glycosyltransferase (LARGE) and LARGE2, further extend this linear carbohydrate branch by synthesizing a unique repeating disaccharide structure [–3-xylose–α1,3-glucuronic acid-β1–]_n_, called matriglycan^25^. This complex carbohydrate is found on the cell surface glycoprotein α-dystroglycan (α-DG). Matriglycan mediates α-DG interactions with LG domains of extracellular matrix (ECM) proteins such as laminins^26–28^ and pikachurin^29–31^, both involved in photoreceptor function. Although POMGnT1 is not directly involved, its activity is essential for efficient synthesis of matriglycan. Since the human EYS molecule has five LG domains, we hypothesized that EYS interacts with matriglycans. In this report, we evaluated this interaction and its significance in *pomgnt1* mutant zebrafish. The data showed that EYS bound to matriglycan *in vitro*. Loss of matriglycan in *pomgnt1* mutant zebrafish resulted in the retention of EYS in the outer nuclear layer and diminished EYS protein near the connecting cilium which resulted in the degeneration of photoreceptors. These results indicate that photoreceptor degenerations in RP25 and RP76 are mechanistically linked in that EYS interacts with the matriglycan moiety of O-mannosyl glycans and that this molecular interaction controls EYS subcellular localization and function to promote photoreceptor survival.

## Results

### EYS interacts with matriglycan moiety of O-mannosyl glycans

**Figure 1:**
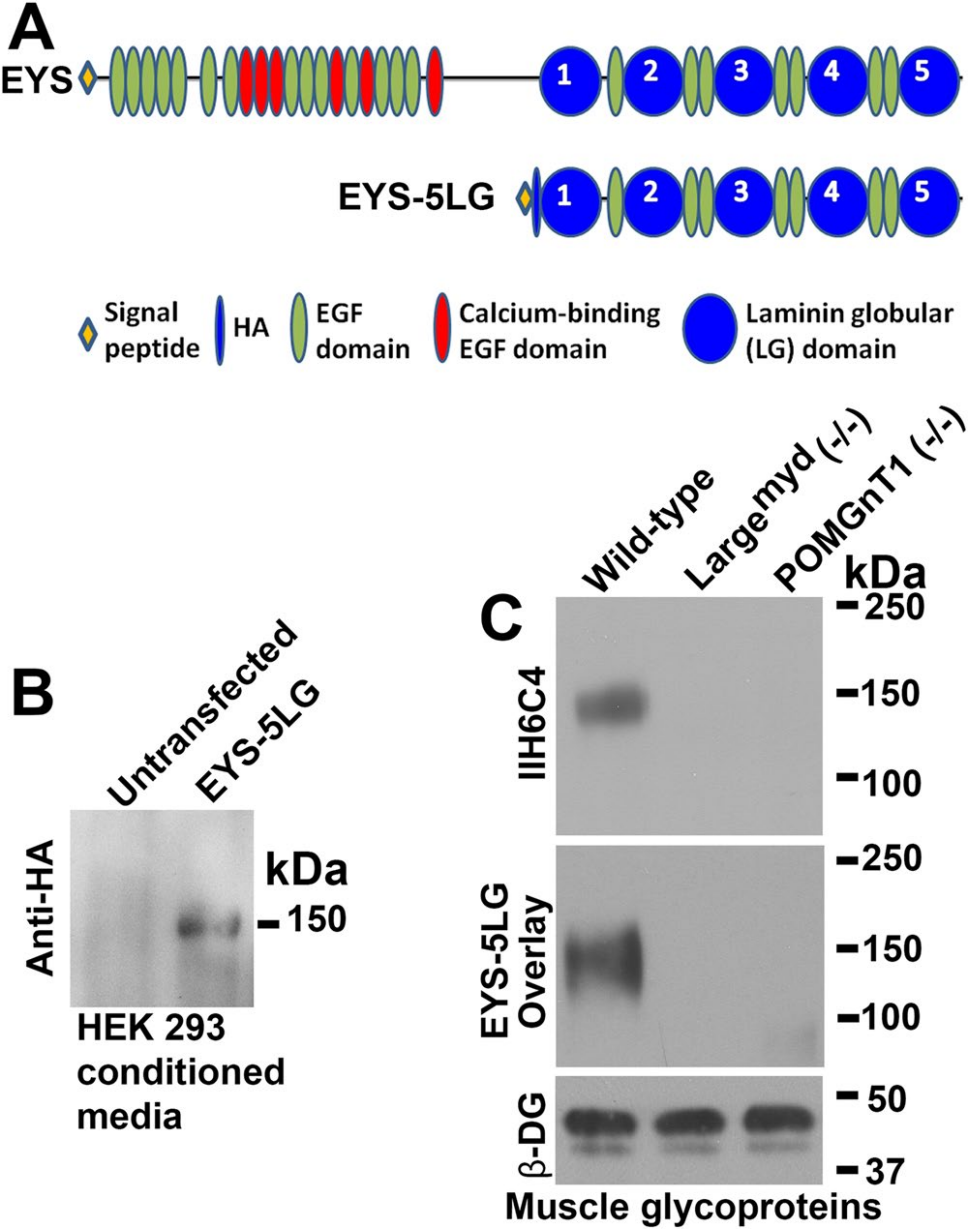
EYS interacts with matriglycans. Human cDNA encoding protein EYS amino acid residues 1862–3165 comprising all 5 LG domains (EYS-5LG) was subcloned into pSecTag2A with N-terminal HA-tag (right after the signal peptide). The cDNA was transfected into HEK293 cells. Conditioned medium containing EYS was collected. EYS Far-Western was carried out on glycoproteins isolated from skeletal muscle of wild-type, LARGE mutant (Large^myd^), and POMGnT1 knockout mice as we have described for laminin Far-Western^21,55–58^. (**A**) EYS-5LG containing 5 LG (blue circle) and 7 EGF (green oval) domains. Location of HA-tag is indicated by the blue oval. (**B**) Recombinant EYS protein was detected at expected 150 kDa in conditioned medium from EYS-transfected cells but not untransfected cells. (**C**) IIH6C4 immunoreactivity was detected at 150 kDa in wild-type but not LARGE mutant and POMGnT1 knockout mice. EYS-5LG binding was detected in the wild-type but not Large^myd^ mice at molecular weight of 150 kDa. Equivalent signal intensities were observed for anti-β-DG, a member of the dystroglycan protein complex. These data indicate that EYS was capable of binding to matriglycan of O-mannosyl glycans.

Matriglycan-binding extracellular matrix proteins such as laminins and pikachurin bind to these glycans via their LG domains. Since human EYS protein has 5 LG domains, we hypothesized that EYS may interact with the matriglycan moiety of O-mannosyl glycans. To evaluate this, we constructed a vector to express a truncated hemagglutinin-epitope (HA) tagged form of EYS protein that contains all of its 5 LG domains (EYS-5LG, Fig. 1A) on the pSecTag2 backbone, transfected the expression vector into HEK293 cells, and collected the conditioned medium. Recombinant EYS-5LG protein was detected at 150 kDa (Fig. 1B) in the conditioned medium from the transfected cells, but not from the non-transfected cells. Next, we carried out an EYS Far-Western blotting assay on glycoproteins isolated from wild-type mouse skeletal muscle lysate using wheat germ agglutinin (WGA)-agarose beads. WGA-binding glycoproteins isolated from POMGnT1 knockout and LARGE mutant (Large^myd^) mice, which are deficient in functional O-mannosyl glycosylation, were used as negative controls. LG domain-binding matriglycan of O-mannosyl glycans are immunoreactive to antibody IIH6C4^26,32^. While glycoproteins isolated from the wild-type showed IIH6C4 immunoreactivity near 150 kDa, glycoproteins isolated from POMGnT1 knockout and Large^myd^ mutant mice did not show detectable immunoreactivity at the same location, as expected^21,33^ (Fig. 1C). Far-Western blotting with EYS-5LG conditioned medium showed that bound EYS was detected at the 150 kDa location with glycoproteins isolated from wild-type but not from the mutant animals (Fig. 1C). As a control, β-DG was detected in glycoproteins from all three samples. These results indicated that EYS was capable of binding to matriglycan of O-mannosyl glycans.

### *pomgnt1* mutation in zebrafish caused diminished expression of matriglycan and EYS binding

**Figure 2:**
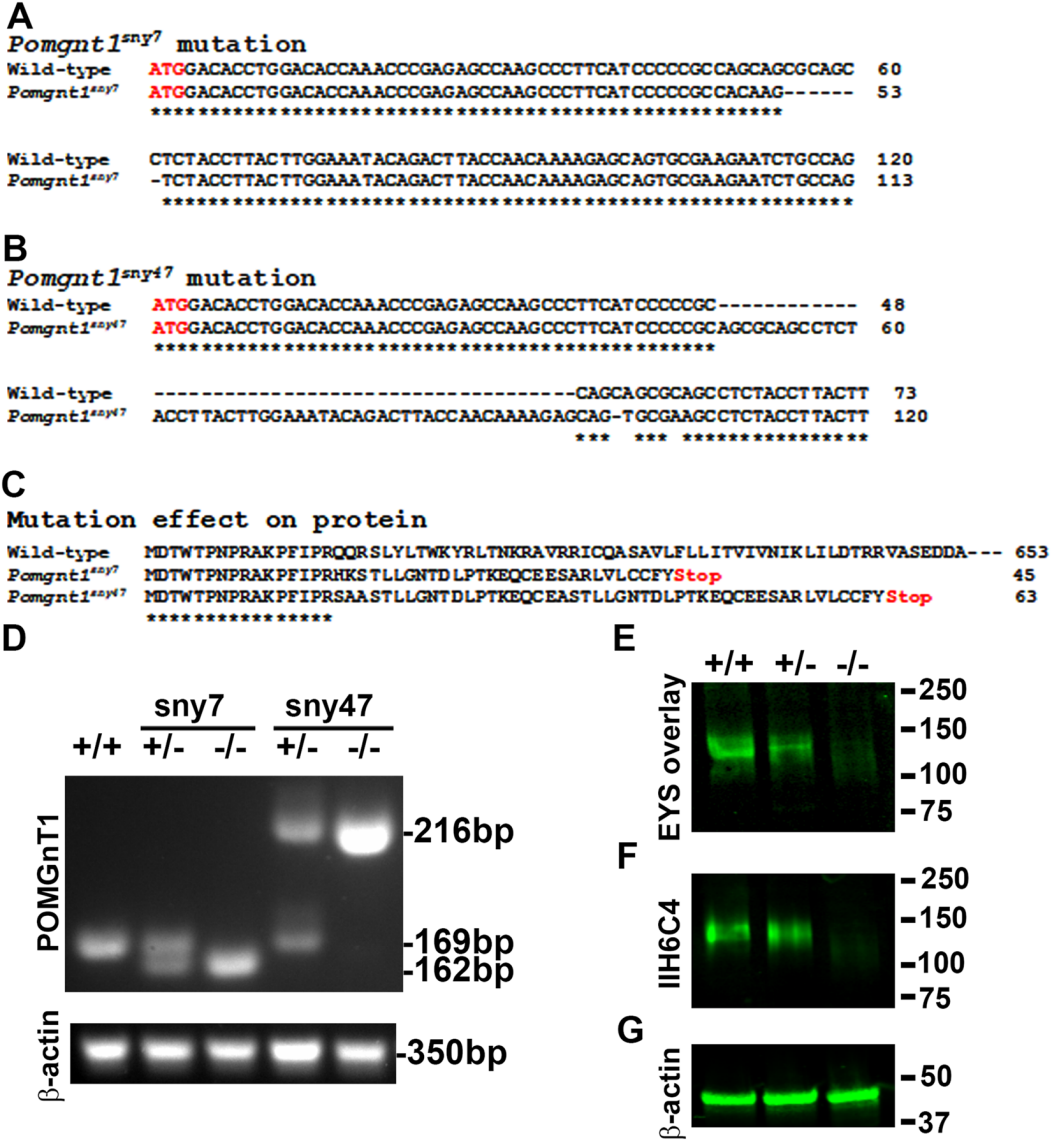
*pomgnt1* mutation in zebrafish caused hypo-O-mannosyl glycosylation and loss of EYS binding. Skeletal muscle lysates were used for IIH6C4 immunoblotting and EYS Far-Western assays. (**A**) Alignment of part of exon 2 sequences, starting with the initiation codon, showing the 7 nt deletion in *pomgnt1*^*sny7*^ mutant.(**B**) Alignment of part of exon 2 sequences showing the multiple mutations in *pomgnt1*^*sny47*^ mutant. (**C**) Alignment of wild-type POMGnT1 protein with the expected truncated POMGnT1 peptides in *pomgnt1*^*sny7*^ and *pomgnt1*^*sny47*^ mutants. (**D**) RT-PCR showing that homozygous mutant animals express only mutant mRNAs. (**E**-**G**) IIH6C4 and β-actin Western blotting and EYS-5LG (see Fig. 1A) Far-Western on *pomgnt1*^*sny7*^ mutants. Note diminished IIH6C4 reactivity and EYS binding in muscle lysate of homozygous *pomgnt1* mutant.

To evaluate the biological significance of EYS-matriglycan interactions, we generated *pomgnt1* mutant zebrafish by CRISPR genome editing. This effort yielded two *pomgnt1* mutant lines, *pomgnt1*^*sny7*^ (Fig. 2A) and *pomgnt1*^*sny47*^ (Fig. 2B). The *pomgnt1*^*sny7*^ allele had a 7-bp deletion (nucleotides 55–61 from the initiation codon) while the *pomgnt1*^*sny47*^ allele featured an insertion of 48-bp between nucleotides 48–49 from the initiation codon, a deletion of 1-bp (nucleotide 52), and a C to A substitution (nucleotide 57), resulting in a net 47-bp insertion. They were frameshift mutations that would result in severely truncated peptides with only 16 of the N-terminal amino acid residues identical to the wild-type POMGnT1 protein, which has 653 amino acid residues (Fig. 2C). Thus, both mutations were expected to be null. Since an antibody against zebrafish POMGnT1 protein was not available, we first validated *pomgnt1* mutant zebrafish by RT-PCR. Wild-type zebrafish produced an expected 169-bp fragment (Fig. 2D). *Pomgnt1*^*sny7*^ and *Pomgnt1*^*sny47*^ mutant mRNAs would generate a 162-bp and a 216-bp RT-PCR fragments, respectively. Indeed, homozygous *pomgnt1* mutant zebrafish expressed only mutant mRNA while heterozygous zebrafish expressed both wild-type and mutant mRNA (Fig. 2D). The band intensity of mutant RT-PCR fragment was similar to the wild-type in heterozygous animals. In addition, the band intensity from homozygous mutants was similar to the wild-type, suggesting that there was no significant level of nonsense-mediated decay of the mutant mRNAs. POMGnT1 deficiency is expected to result in diminished O-mannosyl glycosylation. Thus, we determined whether homozygous *pomgnt1* mutant zebrafish exhibited matriglycan deficiency by Western blotting. Skeletal muscle lysates were immunoblotted with the IIH6C4 antibody. IIH6C4 immunoreactivity was detected in wild-type and heterozygous zebrafish but was diminished in *pomgnt1*^*sny7*^ mutant zebrafish (Fig. 2F). Far-Western blotting with EYS-5LG (Fig. 1A) conditioned medium indicated that binding to EYS was also diminished in *pomgnt1*^*sny7*^ mutant zebrafish when compared to the wild-type and heterozygous samples (Fig. 2E). The internal control β-actin protein immunoreactivity was constant across wild-type, heterozygous and mutant zebrafish samples (Fig. 2G). Similar results were obtained with *pomgnt1*^*sny47*^ mutant zebrafish (data not shown). These results indicated that *pomgnt1* mutant zebrafish exhibited diminished expression of matriglycan.

### **EYS protein was mislocalized in*pomgnt1*mutant retina**

**Figure 3:**
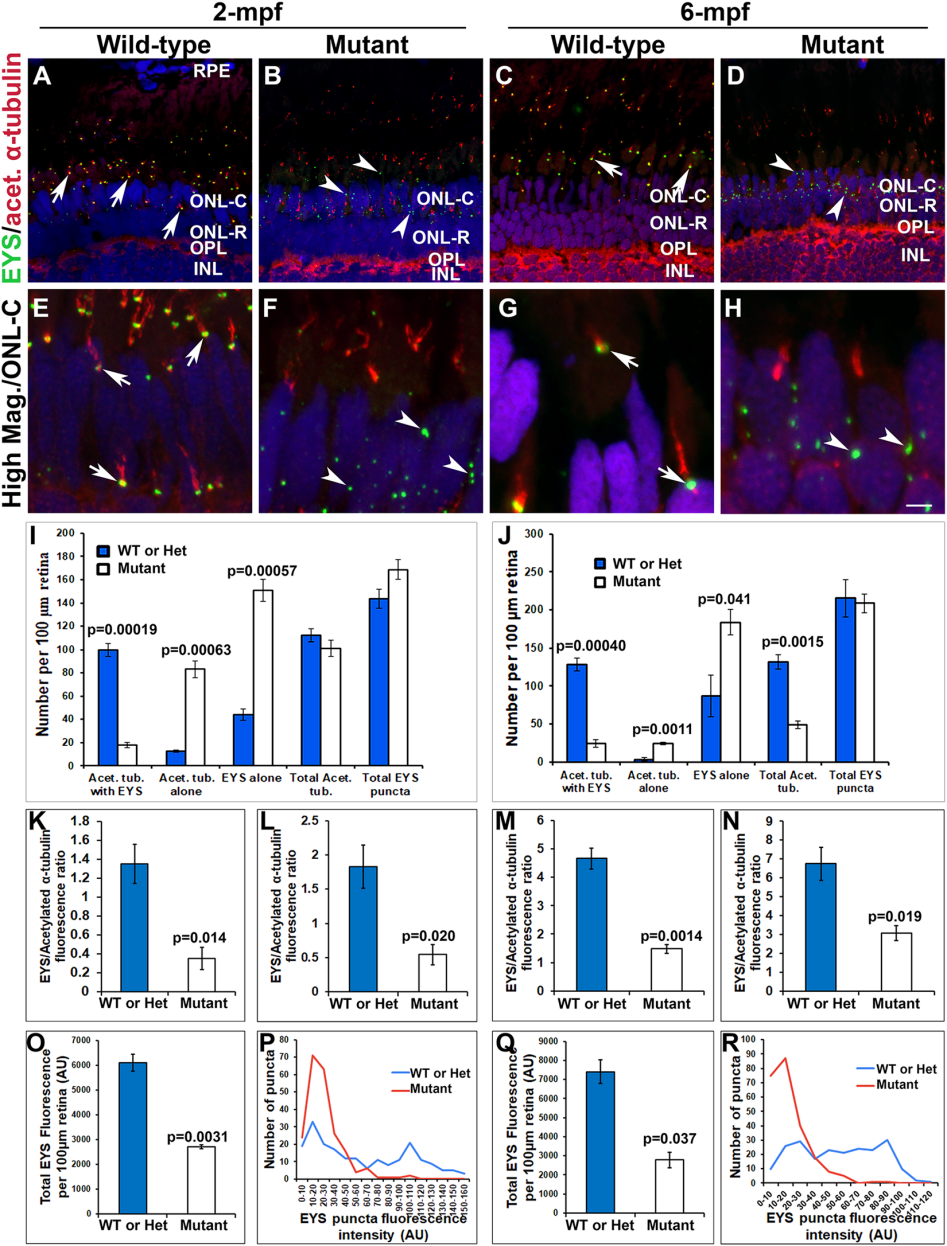
Mislocalization of EYS protein in *pomgnt1* mutant zebrafish retina. Retinal sections from wild-type and *pomgnt1*^*sny7*^ mutant zebrafish were double immunostained with EYS (green fluorescence) and acetylated α-tubulin (red fluorescence). (**A** and **E**) Wild-type retina at 2-mpf. (**C** and **G**) Wild-type retina at 6-mpf. Most EYS-positive puncta in the wild-type retina were associated with basal end of the acetylated α-tubulin reactivity at both 2- and 6-mpf (arrows). (**B** and **F**) Homozygous *pomgnt1*^*sny7*^ mutant retina at 2-mpf. (**D** and **H**) Homozygous *pomgnt1*^*sny7*^ mutant retina at 6-mpf. Most EYS-positive puncta were not associated with acetylated α-tubulin but were localized in the outer nuclear layer at both 2- and 6-mpf (arrowheads). (**I** and **J**) Counting of EYS-acetylated α-tubulin double staining from the outer nuclear layer to the retinal pigment epithelium (RPE) for 2-mpf and 6-mpf retina, respectively. Note that acetylated α-tubulin reactivity associated with EYS puncta was decreased in the mutant, and acetylated α-tubulin not associated with EYS puncta was increased in the mutant. EYS puncta not associated with acetylated α-tubulin was increased in the mutant. ANOVA; post-hoc Student’s t-test with Bonferroni correction.(**K** and **L**) Ratio of EYS and acetylated α-tubulin immunoreactivity for each acetylated α-tubulin-labeled axoneme in rods and cones at 2-mpf, respectively. Note that EYS immunoreactivity at the connecting cilium was reduced by about 5-fold in *pomgnt1* mutant retina. Student’s t-test. (**M** and **N**) Ratio of EYS and acetylated α-tubulin immunoreactivity per acetylated α-tubulin-labeled axoneme in rods and cones at 6-mpf, respectively. Note that EYS immunoreactivity at the connecting cilium was also reduced in the *pomgnt1* mutant retina at 6-mpf. Student’s t-test. (**O** and **Q**) Total EYS immunoreactivity at 2-mpf and 6-mpf, respectively. Total EYS immunoreactivity was reduced in *pomgnt1* mutant retina at both ages. Student’s t-test. (P and R) Histogram representation of total EYS immunoreactivity between wild-type/heterozygous and mutant fish at 2-mpf and 6-mpf, respectively. Note the shift in the number of EYS puncta to lower fluorescence intensities in the *pomgnt1* mutant fish at both ages. Scale bar in H: 5 µm for A-D; 2.5 µm for E-H.

We evaluated EYS protein expression in *pomgnt1* mutant retina by immunostaining of wild-type and mutant sections mounted on the same slide. In wild-type retinas, EYS immunoreactive puncta were observed on the basal end of acetylated α-tubulin, corresponding to the connecting cilium region of photoreceptors as previously reported^12^ (arrows, Fig. 3A,C,E,G). In homozygous *pomgnt1*^*sny7*^ mutant zebrafish retinas, EYS immunoreactivity pattern was significantly altered (Fig. 3B,D,F,H). Unlike the wild-type, very few EYS immunoreactive puncta were observed at the basal end of acetylated α-tubulin. Most were observed within the outer nuclear layer instead (see arrowheads for example). We counted acetylated α-tubulin-positive axonemes associated with visible EYS puncta (Acet. tub. with EYS), acetylated α-tubulin-positive axonemes associated with no visible EYS puncta (Acet. tub. alone), and EYS puncta not associated with axonemes (EYS alone) at the dorsal retina adjacent to the optic nerve head at 2-months post-fertilization (mpf) and 6-mpf (Fig. 3I,J). Homozygous *pomgnt1* mutant retina exhibited significantly reduced number of axonemes associated with EYS puncta but significantly increased number of axonemes not associated with EYS puncta and EYS puncta not associated with axonemes at both 2-mpf and 6-mpf (p < 0.05, Student’s t-test). Similar results were obtained for photoreceptors near the ciliary margin at both 2- and 6-mpf as well as for photoreceptors at 10-dpf (data not shown). Next, we measured EYS immunofluorescence intensity on acetylated α-tubulin-labeled axoneme for rod (Fig. 3K) and cone (Fig. 3L) photoreceptors and normalized to the intensity of acetylated α-tubulin reactivity for 2-mpf retinas. The ratio of EYS/acetylated α-tubulin was significantly reduced at the connecting cilia of both rods and cones in *pomgnt1* mutant retina (p < 0.05, Student’s t-test). Relative EYS expression level between the wild-type and *pomgnt1* mutant retinas was compared by measuring the immunofluorescence intensity of all individual EYS puncta on a confocal image from each animal. Although the numbers of EYS puncta at 2-mpf was similar between the wild-type and mutant animals (Fig. 3I), there were a leftward shift in the histogram of fluorescence intensity of the puncta in the mutants (Fig. 3P), suggesting an overall reduction of EYS immunoreactivity in *pomgnt1* mutant retina. To further evaluate this, we measured the total EYS immunoreactivity at 2-mpf (Fig. 3O). While EYS immunoreactivity was 6100.26 +/−517.55 artificial units (AU)/100 μm of retina (mean +/−SEM) in the wild-type animals, its immunoreactivity was reduced to 2715.55 +/−113.37 AU/100 μm of retina in *pomgnt1* mutant retina (p = 0.0031, Student’s t-test), indicating that EYS protein was reduced in the mutant retina. Similarly, we measured EYS fluorescence intensity in 6-mpf retinas. EYS immunoreactivity at the connecting cilia of both rods and cones was reduced (Fig. 3M,N). Total EYS immunoreactivity was also reduced (Fig. 3Q,R). Similar results were obtained for *pomgnt1*^*sny47*^ mutant animals (data not shown). These results indicated that EYS protein was largely mislocalized as well as reduced in *pomgnt1* mutant retina.

### **Mislocalized EYS protein in*pomgnt1*mutant retina was associated with synaptotagmin-1-positive secretory vesicles**

Some of the mislocalized EYS puncta in the outer nuclear layer of *pomgnt1*^*sny7*^ mutant retina appeared to be over the DAPI-labeled nuclei in the outer nuclear layer (Fig. 3B,F,D,H). However, when 201 EYS immunoreactive puncta that appeared over the DAPI-labeled nuclei were carefully analyzed by orthogonal views with ImageJ, none of them were within DAPI-labeled nuclei, indicating that mislocalized EYS protein was not in the nuclei. To evaluate whether mislocalized EYS in *pomgnt1* mutant retina was localized in other cell organelles, we performed double immunofluorescence staining of EYS antibody with commercially available antibodies against mitochondria marker ATPB, late endosome/lysosome marker VPS33B, and recycling endosome marker Rab11a. EYS immunoreactivity was not co-localized with ATPB (Supplementary Fig. 1), VPS33B (Supplementary Fig. 2), or Rab11a (Supplementary Fig. 3) in both wild-type and *pomgnt1* mutant retina, indicating that mislocalized EYS protein in *pomgnt1* mutant photoreceptors was not located in the mitochondria, late endosome/lysosome, or recycling endosome compartments.

**Figure 4:**
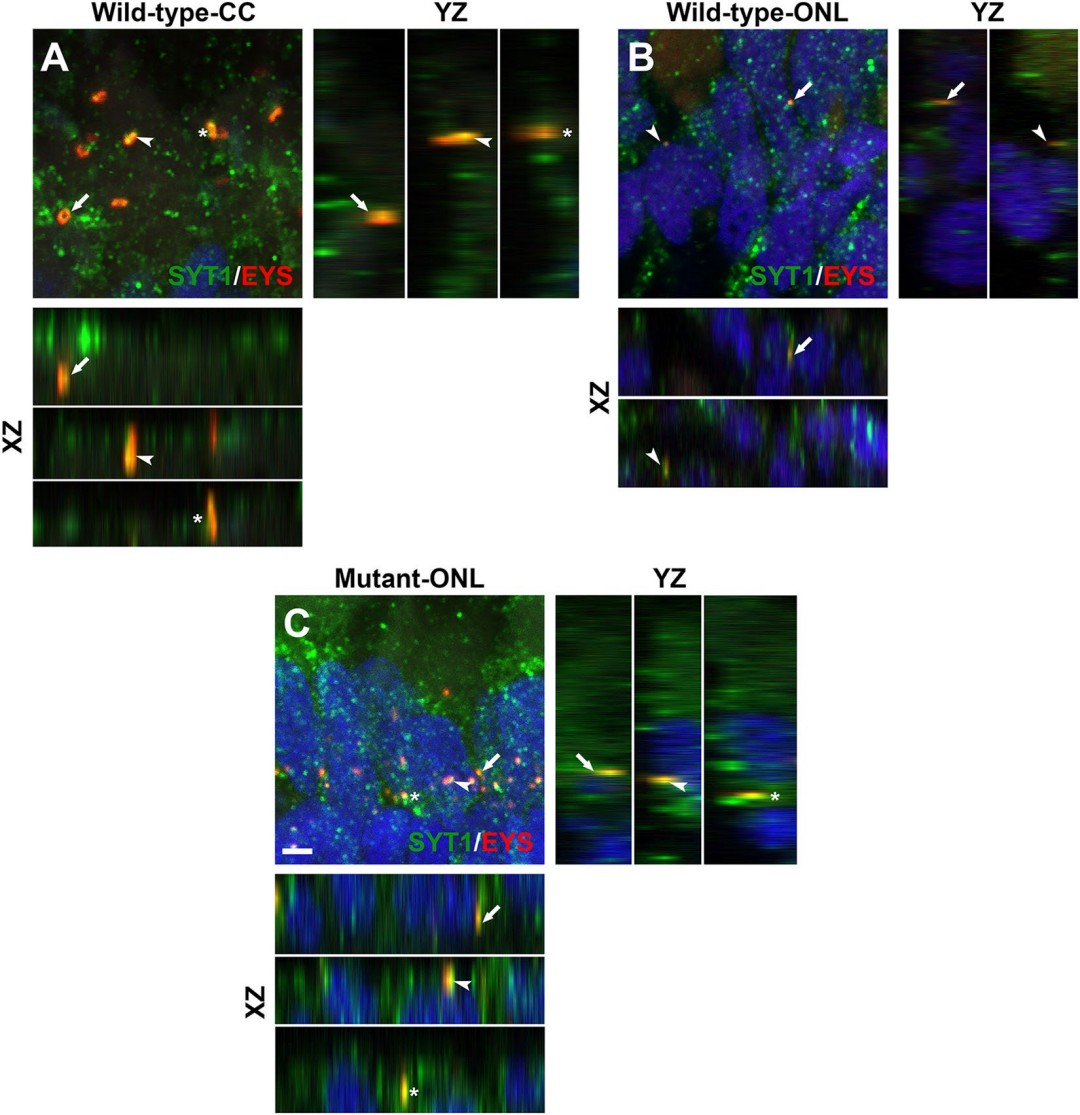
Mislocalized EYS in *pomgnt1* mutant zebrafish retina was co-localized with synaptogamin-1. Retinal sections from 2-mpf zebrafish were double stained with antibodies against EYS (red fluorescence) and synaptotagmin-1 (green fluorescence). (**A**) Wild-type inner/outer segment layers showing connecting cilia (CC). Maximal projection and its orthogonal views of three double stained puncta are shown. EYS-immunoreactivity was co-localized with synaptagmin-1 reactivity. (**B**) Wild-type outer nuclear layer (ONL). Maximal projection and its orthogonal views of two double stained puncta are shown. EYS-immunoreactivity was co-localized with synaptagmin-1 reactivity. (**C**) Homozygous *pomgnt1*^*sny7*^ mutant outer nuclear layer. Maximal projection and its orthogonal views of three double stained puncta are shown. Mislocalized EYS immunoreactive puncta was co-localized with synaptotagmin-1 reactivity. Scale bar in C: 2 µm.

We then evaluated whether mislocalized EYS was in secretory vesicles by double immunofluorescence staining of EYS with synaptotagamin-1. In the wild-type, synaptotagmin-1 immunofluorescence was observed not only as a punctate pattern as expected for secretory vesicular marker, most of the EYS puncta at the connecting cilia were also co-localized with synaptotagmin-1 reactivity (106 out of 111) (Fig. 4A, see arrows, arrowheads, and asterisks for three examples) indicating the presence of synaptotagmin-1 near the connecting cilia. A few EYS puncta could be observed in the outer nuclear layer of wild-type retina as well. Most of these EYS puncta were also reactive to synaptotagmin-1 (24 out of 26) (Fig. 4B), indicating they were localized within secretory vesicles. In *pomgnt1*^*sny7*^ mutant retina, virtually all of the EYS puncta in the outer nuclear layer were immunoreactive to synaptotagmin-1 (99 out of 100) (Fig. 4C, see arrows, arrowheads and asterisks for three examples), indicating that most of the mislocalized EYS within the outer nuclear layer were in secretory vesicles.

### ***pomgnt1*mutation caused photoreceptor degeneration in zebrafish**

**Figure 5:**
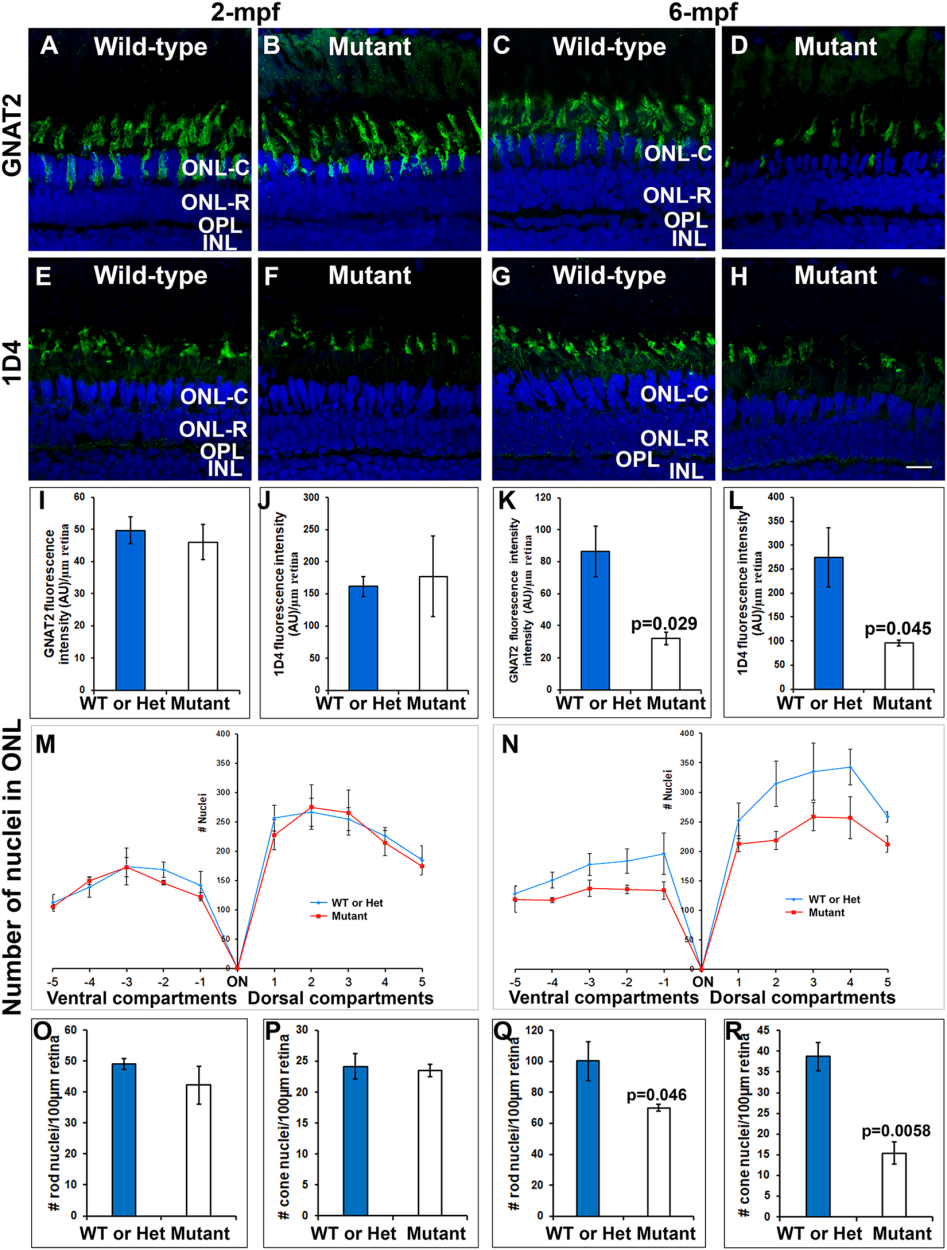
Loss of photoreceptors in *pomgnt1* mutant retinas at 6-mpf but not 2-mpf. Retinal sections from 2-mpf and 6-mpf zebrafish were immunostained with GNAT2 antibody (green fluorescence, **A**–**D**) and 1D4 (green fluorescence, **E**–**H**). The sections were counter-stained with DAPI. To quantify photoreceptors, DAPI fluorescence confocal images of dorsal and ventral retinas from the optic nerve head to the ciliary margin were each divided into 5-equal compartments. Nuclei within the outer nuclear layer of each compartment were counted (**M** and **N**). GNAT2 and 1D4 immunoreactive intensities were measured (**I**–**L**). (**A**) Wild-type GNAT2 immunostaining at 2-mpf. (**B**) Homozygous *pomgnt1*^*sny7*^ mutant GNAT2 immunostaining at 2-mpf showing similar immunoreactivity compared to wild-type. (**C**) Wild-type GNAT2 immunostaining at 6-mpf. (**D**) Homozygous *pomgnt1*^*sny7*^ mutant GNAT2 immunostaining at 6-mpf showing reduced immunoreactivity compared to wild-type. (**E**) Wild-type 1D4 immunostaining at 2-mpf. (**F**) Homozygous *pomgnt1*^*sny7*^ mutant 1D4 immunostaining at 2-mpf showing similar immunoreactivity compared to the wild-type. (**G**) Wild-type 1D4 immunostaining at 6-mpf. (**H**) Homozygous *pomgnt1*^*sny7*^ mutant 1D4 immunostaining at 6-mpf showing reduced immunoreactivity compared to the wild-type. (**I**) GNAT2 immunofluorescence intensity quantification of wild-type and mutant fish at 2-mpf. There was no significant difference between wild-type and mutant retinas. (**J**) 1D4 immunofluorescence intensity quantification of wild-type and mutant fish at 2-mpf. There was no significant difference between wild-type and mutant retinas. (**K** and **L**) GNAT2 and 1D4 immunofluorescence intensity (artificial units, AU) in the mutant retina was reduced at 6-mpf. Student’s t-test. (**M**) Nuclei counting of outer nuclear layer at 2-mpf. There was no significant difference between wild-type and mutant retinas. (**N**) Nuclei counting of outer nuclear layer at 6-mpf. Nuclei within the outer nuclear layer in the mutant were reduced in number compared to wild-type. P = 0.00026; repeated measures ANOVA. (**O** and **P**) 2-mpf nuclei count in the ONL-R and ONL-C retinal layers, respectively. There was no significant difference between wild-type and homozygous *pomgnt1*^*sny7*^ mutants. (**Q** and **R**) 6-mpf nuclei count in the ONL-R and ONL-C retinal layers, respectively. Note the significant reduction of nuclei in both ONL-R and ONL-C in homozygous *pomgnt1*^*sny7*^ mutants at 6-mpf. Together, these results indicated photoreceptor degeneration in mutant retina. Student’s t-test. Scale bar in H: 5 µm.

Since EYS-deficiency causes photoreceptor degeneration in zebrafish^12–14^, diminished EYS near the connecting cilia in *pomgnt1* mutant zebrafish may result in photoreceptor degeneration. The number of acetylated α-tubulin-labeled axonemes in *pomgnt1*^*sny7*^ mutant retinas was similar to the wild-type retina at 2-mpf (Fig. 3I) but was reduced at 6-mpf when compared to the wild-type (p = 0.0015, Student’s t-test, Fig. 3J), suggesting loss of photoreceptors at 6-mpf. To further evaluate photoreceptor degeneration, we carried out immunostaining with G protein subunit alpha transducin 2 (GNAT2) antibody (a general cone marker) and antibody 1D4 (a long double cone marker^34^) (Fig. 5A–H). At 2-mpf, GNAT2 and 1D4 immunoreactivity pattern in homozygous *pomgnt1*^*sny7*^ mutant zebrafish retina was very similar to the wild-type zebrafish (Fig. 5A,B,E,F). GNAT2 and 1D4 immunofluorescence intensity in the mutant retina was also very similar to the wild-type retina (Fig. 5I,J). At 6-mpf, however, GNAT2 immunoreactivity in homozygous *pomgnt1*^*sny7*^ mutant zebrafish was reduced (compare Fig. 5D,C,K). 1D4 immunoreactivity was also significantly reduced in homozygous *pomgnt1*^*sny7*^ mutant zebrafish when compared to the wild-type zebrafish at 6-mpf (Fig. 5H,G,L).

Next, we counted DAPI-labeled nuclei in the outer nuclear layer of mutant retinal sections (Fig. 5M,N). Images for dorsal and ventral retina from the optic nerve head to the ciliary margin were divided into 5-equal compartments, respectively. Nuclei within the outer nuclear layer of each compartment were counted. Nuclear density in homozygous *pomgnt1* mutant zebrafish was similar to the wild-type at 2-mpf (Fig. 5M). However, the nuclear density was significantly reduced in *pomgnt1*^*sny7*^ mutant retina at 6-mpf (Fig. 5N, p = 0.00026, repeated measures ANOVA), suggesting a loss of photoreceptors in *pomgnt1* mutant retinas at 6-mpf. We also counted the nuclei in the rod outer nuclear layer (ONL-R) and cone outer nuclear layer (ONL-C) separately from dorsal retinal images obtained near the optic nerve head. There was no significant difference in the number of ONL-R and ONL-C nuclei between the wild-type and mutant fish at 2-mpf (Fig. 5O and P); however, those numbers were significantly reduced in the 6-mpf mutant fish (Fig. 5Q,R), further supporting photoreceptor degeneration in *pomgnt1* mutants.

**Figure 6:**
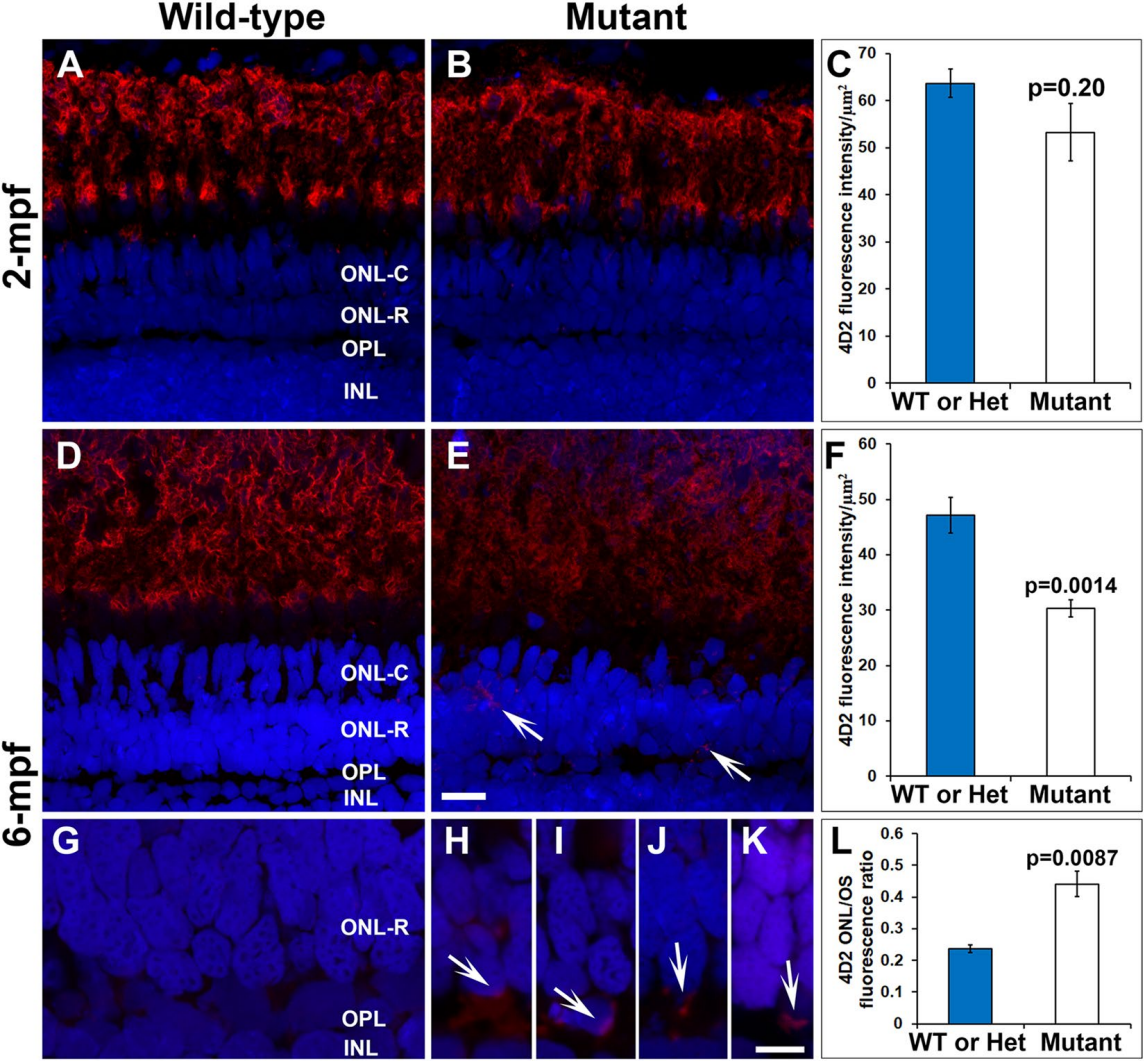
Reduction and mislocalization of rhodopsin in *pomgnt1* mutant retina. Retinal sections from 2-mpf and 6-mpf zebrafish were immunostained with antibody 4D2 (red) and counter-stained with DAPI (blue). (**A**) 4D2 immunostaining of wild-type retina at 2-mpf. (**B**) 4D2 immunostaining of homozygous *pomgnt1*^*sny7*^ mutant retina at 2-mpf. (**C**) Quantification of 4D2 fluorescence intensity at 2-mpf. There was no significant reduction in 4D2 intensity in the mutant fish at this age. Student’s t-test. (**D**) 4D2 immunostaining of wild-type retina at 6-mpf. (**E**) 4D2 immunostaining of homozygous *pomgnt1*^*sny7*^ mutant retina at 6-mpf. Note the reduction in 4D2 fluorescence intensity. Note the mislocalization of 4D2 immunoreactivity to the ONL-R (arrows). (**F**) Quantification of 4D2 fluorescence intensity at 6-mpf. There was a significant reduction in 4D2 intensity in the mutant fish at this age. Student’s t-test. (**G**) High mag of wild-type 4D2 staining at 6-mpf. Fluorescence intensity of 4D2 in the ONL-R was low. (**H**–**K**) High mag of homozygous *pomgnt1*^*sny7*^ mutant 4D2 staining at 6-mpf. Note the mislocalization of 4D2 immunoreactivity to the ONL-R layer (arrows). (**L**) Ratio of 4D2 fluorescence intensity of outer nuclear layer/outer segment layer (ONL/OS). 4D2 ONL/OS fluorescence ratio was increased in the mutants. Student’s t-test. Scale bar in E: 5 µm for A, B, D and E; scale bar in K: 5 µm for G-K.

We then evaluated rod photoreceptors by immunostaining with the rhodopsin antibody (4D2). 4D2 immunoreactivity was similar between the wild-type and mutant retinas at 2 mpf (Fig. 6A–C). At 6-mpf, immunoreactivity to 4D2 in *pomgnt1* mutant animals (4 *pomgnt1*^*sny7*^ and 1 *pomgnt1*^*sny47*^ animals) was reduced (Fig. 6D–F). Furthermore, 4D2 fluorescence intensity in the outer nuclear layer of wild-type animals was similar to background intensity levels (Fig. 6G). In *pomgnt1* mutant animals, however, 4D2 reactivity was frequently found in the outer nuclear layer (Fig. 6H–K). Additionally, the ratio of 4D2 immunofluorescence intensity in the outer nuclear layer and outer segment layer in *pomgnt1* mutant was increased, indicating the mislocalization of rhodopsin in the mutant photoreceptors (Fig. 6L). Together, these results suggest the degeneration of rod photoreceptors in *pomgnt1* mutant animals in an age dependent manner.

## Discussion

Our results indicated that EYS was capable of binding to the matriglycan moiety of O-mannosyl glycans. Diminished matriglycan in *pomgnt1* mutant zebrafish resulted in mislocalization of EYS protein. Instead of being located near the connecting cilium as in the wild-type, most EYS protein in the mutant photoreceptors was localized in synaptotagmin-1-positive secretory vesicles. The photoreceptor density of *pomgnt1* mutant retina was similar to the wild-type at 2-mpf but was significantly reduced at 6-mpf. Together, these results demonstrate that EYS interacts with matriglycan of O-mannosyl glycans and that this interaction is essential for EYS localization near the connecting cilia and photoreceptor survival.

α-DG is a widely expressed glycoprotein that binds to the transmembrane β-DG which, in turn, interacts with the cytoskeleton. As a cell-surface receptor, α-DG interacts with LG domains of several ECM proteins including laminins^26–28^, agrin^35^, perlecan^36^, neurexin-1^37^, Slit2^38^, and pikachurin^29–31^. These interactions are mediated by matriglycan, a linear chain of repeating disaccharides linked to O-linked mannose on α-DG, [-3-xylose–β1,3-glucuronic acid-α1,-]_n_^25,32^. Our data showed that recombinant C-terminal half of EYS protein containing all of its LG domains was capable of binding to matriglycans. We do not know whether EYS interacts with matriglycans on α-DG or on another unidentified glycoprotein in zebrafish photoreceptors. Early lethality of dystroglycan mutant zebrafish^39^ prevents evaluation of the role of α-DG on EYS expression. A dystroglycan conditional mutant zebrafish line would be helpful to determine whether α-DG is required for EYS protein localization and function.

Matriglycan is synthesized by a Golgi enzyme called like-glycosyltransferase (LARGE)^40^ or LARGE2^41^. Although POMGnT1 is not directly involved, its enzymatic activity is required for efficient synthesis of matriglycan^42,43^. Thus, *pomgnt1* mutant zebrafish is expected to result in hypo-O-mannosyl glycosylation and diminished matriglycan. Indeed, *pomgnt1* mutant zebrafish exhibited loss of reactivity to IIH6C4 antibody, which resulted in diminished EYS binding. These results indicate that EYS protein interacts with matriglycans. All matriglycan-binding proteins have multiple LG domains except for Slit2, which has a single LG domain. For example, of the five LG domains in laminin α2, LG 4 and 5 mediate matriglycan interactions while LG 1–3 are involved in binding to integrins^44^. Likewise, we speculate that LG domains 4–5 of EYS may also be involved in binding to matriglycan. Far-Western blot analysis of EYS-5LG fragments with serial deletion of LG domains will identify the LG domains involved in matriglycan binding.

Most EYS mutations found in RP25 patients are frameshift or nonsense mutations. However, some missense mutations have been found within LG domains such as p.D2767Y and p.D3028Y mutations in LG domain 4 and 5^45,46^. Protein alignment analysis indicates that D2767 and D3028 of EYS correspond to D2807 of laminin α2 LG domain 4, which has been shown to be essential for coordinating Ca^++^ ion binding to the glucuronic acid-β1,3-xylose disaccharide repeat of matriglycan^47^. It will be interesting to evaluate the impact of these mutations on EYS-matriglycan interactions.

Our results are consistent with the idea that POMGnT1 is a Golgi enzyme participating in the O-mannosyl glycosylation pathway. It is of interest to note that POMGnT1 protein immunoreactivity in the mouse was proposed to be localized at the basal body and daughter centriole of photoreceptors^19^. Whether POMGnT1 also functions at the basal body and daughter centriole in addition to participating in the O-mannosyl glycosylation pathway will need to be further evaluated.

We previously showed that EYS immunoreactivity was highly enriched near the connecting cilium in zebrafish^12^, which was confirmed by an independent study^14^. The current study revealed that EYS-matriglycan interaction was important for proper localization of the EYS protein. Instead of localizing near the connecting cilium, EYS protein was largely found within the outer nuclear layer in *pomgnt1* mutant retina. EYS protein is presumed to be synthesized in the ER and trafficked to the Golgi apparatus before being secreted to the connecting ciliary region. Most of the mislocalized EYS puncta in *pomgnt1* mutant retina were co-localized with synaptotagmin-1-positive puncta (a secretory vesicle/v-SNARE marker), indicating that they were in secretory vesicles.

The finding of hypomorphic POMGnT1 mutations in retinitis pigmentosa (RP76) patients^19,20^ supports that POMGnT1 deficiency causes photoreceptor degeneration. Mouse models of O-mannosyl glycosylation deficiency, POMGnT1^21^, fukutin^48^, and Large^myd^^33,49^ mutant mice, exhibit multiple retinal defects including a thinner retina, disrupted inner limiting membrane, reactive gliosis, and defective ribbon synaptic transmission. Mice deficient in dystroglycan^50,51^ or matriglycan-binding ECM proteins pikachurin^29,30^ and β2 or γ3 subunit-containing laminins^52–54^ recapitulates some of these retinal phenotypes. However, there is no evidence that O-mannosyl glycans are required for photoreceptor survival in the mouse. We have evaluated 1.5-year old POMGnT1 knockout mice and found no evidence of progressive loss of photoreceptors or increased cell death (Yu, M. and Hu, H., unpublished results). In this report, *pomgnt1* mutant zebrafish exhibited an age-dependent loss of photoreceptors. These results indicated that *pomgnt1* mutation in zebrafish caused photoreceptor degeneration, strongly supporting that POMGnT1 mutation is the root cause of photoreceptor degeneration in RP76.

It is intriguing that the mouse does not have an EYS locus and that photoreceptors do not degenerate in POMGnT1 mutant mice. It is possible that another matriglycan-binding protein functions in place of EYS, or alternatively, mouse photoreceptors have adapted to the loss of EYS via cellular and molecular changes such that they no longer require EYS to survive. Given that POMGnT1 knockout mice do not exhibit photoreceptor degeneration, we speculate that mouse photoreceptors may have made structural or molecular adaptations to the loss of EYS. Understanding why mouse photoreceptors survive without EYS may be useful for therapeutic development to treat RP25 patients.

This study showed that the matriglycan binds to EYS. Diminished matriglycan in *pomgnt1* mutant zebrafish resulted in a failure of EYS protein secretion to the connecting cilium region and mislocalization of EYS-enriched secretory vesicles in the outer nuclear layer. Without sufficient EYS at the connecting cilium, the photoreceptors die in an age-dependent manner, as in EYS-deficient zebrafish. These data indicate that POMGnT1 supports photoreceptor survival by O-mannosyl glycosylation, which in turn is required for targeting EYS protein to the connecting cilium region. They are consistent with the idea that POMGnT1 contributes to photoreceptor survival by participating in functional O-mannosyl glycosylation to regulate EYS protein localization. Thus, RP25 and RP76 are mechanistically linked by molecular interactions of EYS protein with the matriglycan, whose biosynthesis is dependent on POMGnT1.

## Materials and Methods

### Zebrafish maintenance

Zebrafish (AB/Tubingen strain) were grown in a recirculating water system (pH 6.6–7.4) at 26–28.5 °C with a daily light cycle of 14 hours of light and 10 hours of darkness. They were fed once daily with Gemma Micro (Skretting, Tooele, Utah). All experiments on zebrafish animals were in accordance with the National Institute of Health guidelines and approved by the Institutional Animal Care and Use Committee at the State University of New York Upstate Medical University.

### **Generation of*pomgnt1*mutant lines**

The zebrafish *pomgnt1* locus is located on chromosome #6. Alignment of zebrafish and human POMGnT1 revealed that 79% of amino acid sequences were identical between the two orthologs. To determine the impact of POMGnT1 deficiency on EYS and photoreceptor health, we used clustered regularly interspaced short palindromic repeats (CRISPR)/Cas9 technology to generate *pomgnt1* mutants.

Two gRNAs targeting exon 2 of the *pomgnt1 locus*, GTAGAGGCTGCGCTGCTGGC, and GGTAGAGGCTGCGCTGCTGG were synthesized at GenScript (Piscataway, NJ) and co-injected with SpCas9 protein (New England Biolabs) at 1–2 cell stage of embryos. Tail-fin clips from surviving zebrafish were used to amplify a fragment spanning the gRNA target sites with primers forward TGAAGAAGACGTGATCAAAGG and reverse TCATGTACTTTTGGGGATTTCA, which produce a 338 nt fragment by PCR. Animals with abnormal sized amplicons were crossed with wild-type animals to obtain F1 generation. F1 animals were then screened by PCR with the same primers. Abnormal sized bands were then extracted from agarose gels and sequenced to identify the mutation. This effort yielded two mutant lines containing a deletion of 7 nucleotides and a net insertion of 47 nucleotides. We have named these mutant alleles as *pomgnt1*^*sny7*^ and *pomgnt1*^*sny47*^ in accordance with the convention on zebrafish mutant line designation. Both mutations occur within exon 2 and are expected to disrupt the reading frame resulting in the loss of POMGnT1 protein (Fig. 2). Thus, these frameshift mutations are presumed nulls.

The phenotypes in homozygous *pomgnt1*^*sny7*^ and *pomgnt1*^*sny47*^ mutant zebrafish were indistinguishable. Thus, the results from both lines were combined in this report. A total of 13 homozygous *pomgnt1*^*sny7*^ mutant fish including 1 at 10 days post fertilization (dpf), 5 at 2 month post fertilization (mpf), 3 at 4 mpf, 4 at 6 mpf and a total of 5 homozygous *pomgnt1*^*sny47*^ mutant fish including 1 at 10 dpf, 3 at 2 mpf, 1 at 6 mpf and their respective body weight matched wild-type or heterozygous controls were used in the experiments.

### RT-PCR

Skeletal muscle of the body walls from 2-month old zebrafish was used to extract total RNA using RNeasy Plus Mini Kit (QIAGEN). POMGnT1 RT-PCR was carried out with the iTaq Universal SYBR Green One-Step Kit (Bio-Rad) with primers forward AGGTGTTGTGATCAGGAGCAG and reverse ACAGCACTAGCCTGGCAGAT, which generate a 169-bp amplicon for wild-type mRNA. Internal control β-actin RT-PCR primers were forward TTCCTGGGTATGGAATCTTGC and reverse GGTGGCAACAGTTCTGTTTAG, which give a 350-bp amplicon.

### Western blotting

For mouse skeletal muscle samples, protein was extracted with radio-immuno precipitation assay (RIPA) buffer (50 mM Tris, pH 8.0, 150 mM NaCl, 1.0% NP-40, 0.5% sodium deoxycholate, 0.1% SDS) and protease inhibitor cocktail. Ten mg of total protein was used to isolate glycoproteins by wheat germ agglutinin (WGA)-agarose as described before^21^ and separated on SDS-PAGE and transferred to PVDF membranes. After blocking with Tris-buffered saline (50 mM Tris, pH 7.4, 150 mM NaCl) containing 1% BSA, the membrane was incubated with IIH6C4 (Santa Cruz, Cat# sc-73586, 1:400 dilution), anti-β-dystroglycan (Abcam, Cat# ab49515), or anti-β-actin (Cell Signaling Technology, Cat# 8457, 1:500 dilution) antibodies in TBS with 0.2% Tween-20 and 1% BSA overnight at 4 °C with gentle shaking. After washing with TBST (TBS with 0.1% Tween 20) three times, the membrane was incubated with horse radish peroxidase-conjugated secondary antibody in TBST for 2 hrs at room temperature. The membrane was then washed three times in TBST and once with TBS and was developed with SuperSignal West Pico Chemiluminescent substrate.

For zebrafish skeletal muscle samples, lysates were prepared from skeletal muscle of the body walls. Lysate proteins were separated on SDS-PAGE, transferred to PVDF membrane, and incubated with IIH6C4 and β-actin antibodies as above. Blotting signal was detected with DyLight Fluor secondary antibodies and visualized with Odyssey CLx (Li-Cor Biosciences).

### EYS Far-Western Blot

cDNA fragment encoding human EYS protein amino acid residues 1862–3144 comprising all 5 laminin G domains was synthesized and subcloned into pSecTag2A with N-terminal HA-tag (GenScript). The expression vector was transfected into HEK293 cells. Conditioned medium was collected 2 days after transfection.

For eyes shut homolog Far-Western assay on mouse muscle lysates, WGA-bound glycoproteins were separated on SDS-PAGE and transferred to PVDF membranes. The PVDF membrane was incubated with 3% BSA in Tris-buffered saline (50 mM Tris, pH 7.4, 150 mM NaCl, 1 mM CaCl_2_, and 1 mM MgCl_2_) for 30 minutes and then eyes shut homolog-conditioned medium overnight at 4 °C. Immunodetection of bound eyes shut homolog with anti-HA was identical to the above procedure with the exception that all buffers contained 1 mM CaCl_2_ and 1 mm MgCl_2_. After washing with TBST, the membrane was incubated with a rabbit antibody against laminin (1:2000) for 2 hrs and washed with TBST. The membrane was then incubated with goat anti-rabbit IgG conjugated with horseradish peroxidase (1:3000) for 45 min. After extensive washing with TBST, the signal was visualized with SuperSignal West Pico Chemiluminescent substrate.

For eyes shut homolog Far-Western assay on zebrafish samples, muscle lysate proteins were separated on SDS-PAGE and transferred to PVDF membrane. Far-Western was performed as above but the blotting signal was detected with DyLight Fluor secondary antibodies and visualized with Odyssey CLx (Li-Cor Biosciences).

### Immunofluorescence staining

Whole heads were embedded in OCT medium and cryo-sectioned along the dorso-ventral plane. Sections were mounted onto Fisherbrand Superfrost plus slides. The sections were fixed in 4% paraformaldehyde, permeabilized with 0.1% Triton X-100 in phosphate buffer, and blocked with 3% bovine serum albumin (BSA) in 0.1 M phosphate buffer (PB) at room temperature for 1 h in a humidified environment. Primary antibodies against GNAT2 (MBL International, Cat#PM075, 1:400), EYS (Novus Biological, Cat# NBP1–90038, 1:300), acetylated α-tubulin (Sigma, Cat#T6793, 1:1000), long double cone (1D4, Abcam, Cat#AB5417, 1:1000), rhodopsin (4D2, Abcam, Cat#AB98887, 1:300), ATPB (Abcam, Cat# AB197904, 1:300), ARF6 (LifeSpan, Cat#LS-B5846, 1:100), SNAP-25 (Abcam, Cat#AB5666, 1:100), VPS33B (ProteinTech, Cat#12195-1-AP, 1:100), Rab11a (LifeSpan, Cat#LS-C93526, 1:300), and synaptotagmin-1 (Lifespan Biosciences, Cat#LS-B5899-50, 1:100) were applied overnight at 4 °C. After washing with PB containing 0.1% Triton X-100, the sections were incubated with appropriate FITC-conjugated anti-rabbit IgG (Jackson ImmunoResearch, 1:300) or RITC-conjugated anti-mouse IgG (Jackson ImmunoResearch, 1:300) for 2 hrs at room temperature in a humidified chamber. Counter-staining with 4′,6-diamidino-2-phenylindole (DAPI) was used to visualize the nuclei. After washing with PB three times, the sections were covered with VECTASHIELD Antifade Mounting Medium (Vector Laboratories, Cat# H-1000) by coverslips. To visualize fluorescence, a Zeiss Axioskop epifluorescence microscope and a Zeiss confocal microscope system (Zeiss LSM 780 confocal) were used. Epifluorescence images were captured with a mono 12-bit camera and QCapture Pro 6.0 (QImaging).

To evaluate the co-localization of immunofluorescence using orthogonal projections, Z-series were acquired with a Zeiss LSM780 confocal microscope. Images were exported to ImageJ. Max projection and orthogonal views were generated.

### Semi-quantitative analyses of photoreceptor nuclei numbers and immunofluorescence staining signals

For counting nuclei in the outer nuclear layer, retinal sections were stained with DAPI. Confocal images covering the entire dorsal and ventral retina from the optic nerve head to the ciliary margin were obtained and exported to ImageJ for quantification. The outer nuclear layer from the optic nerve head to the ciliary margin in each image was divided into 5 lengthwise equal compartments. Nuclei in the outer nuclear layer within each compartment were then counted with ImageJ software. A repeated-measures ANOVA was performed in SPSS (IBM) to determine statistical significance using a 0.05 alpha level. Post-hoc comparisons were done by Student’s t-test with Bonferroni correction.

For quantification of EYS association with axoneme, EYS-positive puncta associated with acetylated α-tubulin-positive axoneme, EYS-positive puncta not associated with acetylated α-tubulin-positive axoneme, and acetylated α-tubulin-positive axoneme not associated with EYS puncta, were counted from confocal images from dorsal retina near the optic nerve head. Data were analyzed by ANOVA. Post-hoc comparisons were done by Student’s t-test with Bonferroni correction.

For quantification of EYS immunoreactive intensity at the basal end of acetylated α-tubulin-labeled axoneme, confocal images from the dorsal retina near the optic nerve head of 2-mpf zebrafish were captured with fixed acquisition settings for all samples and exported to ImageJ. Individual acetylated α-tubulin-positive axonemes were manually selected, and their fluorescence intensities were measured. Next, the EYS puncta associated with the acetylated α-tubulin-positive axoneme were also manually selected, and their respective fluorescence intensities were recorded. Background fluorescence was subtracted from both axoneme and EYS intensity measurements. The ratio of EYS/acetylated α-tubulin of each axoneme was calculated. Data were analyzed by Student’s t-test.

To evaluate total EYS protein level in *pomgnt1* mutant retina at 2-mpf, confocal images were exported to ImageJ. All EYS immunoreactive puncta were selected using the selection tool, and fluorescence intensity was measured. Following background fluorescence subtraction, total cumulative EYS fluorescence was calculated and normalized to retinal length. Data were analyzed by Student’s t-test.

For quantification of GNAT2 and 1D4 immunofluorescence intensity, confocal images from the dorsal retina near the optic nerve head were exported to ImageJ. Fluorescence intensity in the entire outer segment layer was measured. Back ground fluorescence intensity was then subtracted. Data were analyzed by Student’s t-test.

## Supplementary information

Supplementary Information.
