# Supplementary material for: Eyes shut homolog (EYS) interacts with matriglycan of O-mannosyl glycans whose deficiency results in EYS mislocalization and degeneration of photoreceptors
Source: Sci Rep. 2020 May 8;10:7795. doi: 10.1038/s41598-020-64752-4 (PMC7210881; doi:10.1038/s41598-020-64752-4)

Supplementary Data

Eyes shut homolog (EYS) interacts with matriglycan of O-mannosyl glycans whose deficiency results in EYS mislocalization and degeneration of photoreceptors

Yu Liu[#], Miao Yu[#], Xuanze Shang, My Hong Hoai Nguyen[1], Shanmuganathan Balakrishnan, Rachel Sager, and Huaiyu Hu[*]

Center for Vision Research, Departments of Neuroscience and Physiology and of Ophthalmology and Visual Sciences, Upstate Medical University, Syracuse, NY 13210

[1]Current address: Department of Biological Sciences, State University of New York at Plattsburgh, 101 Broad St., Plattsburgh, New York 12901

[#]These authors contributed equally to this work.

[*]Corresponding author

Supplementary Figure 1. Mislocalized EYS in *pomgnt1* mutant photoreceptors was not localized with mitochondria.

Cryosections of zebrafish eyes were double stained with anti-EYS (red) and with antibody against mitochondrial marker ATPB (green). The sections were counter-stained with DAPI to visualize nuclei (blue).

(A) Wild-type. In the wild-type retina, ATPB antibody labeled the ellipsoid region of photoreceptors with EYS immunoreactivity observed on the apical side of ATPB reactivity (arrows).

(B) Homozygous *pomgnt1*^{sny7} mutant. Most EYS immunoreactive puncta were localized in the outer nuclear layer. Some appeared to be over the ATPB immunoreactive domain (arrowheads)

(C-G) Mutant. Max projection image and its orthogonal views of EYS puncta that appeared to be over the ATPB immunoreactive domains. Of 81 EYS immunoreactive puncta that appeared to overlap with ATPB immunoreactivity, none were within the ATPB immunoreactive domain, indicating that mislocalized EYS were not within mitochondria.

Scale bar in A: = 4.36 μm for A-B, 2 μm for C.

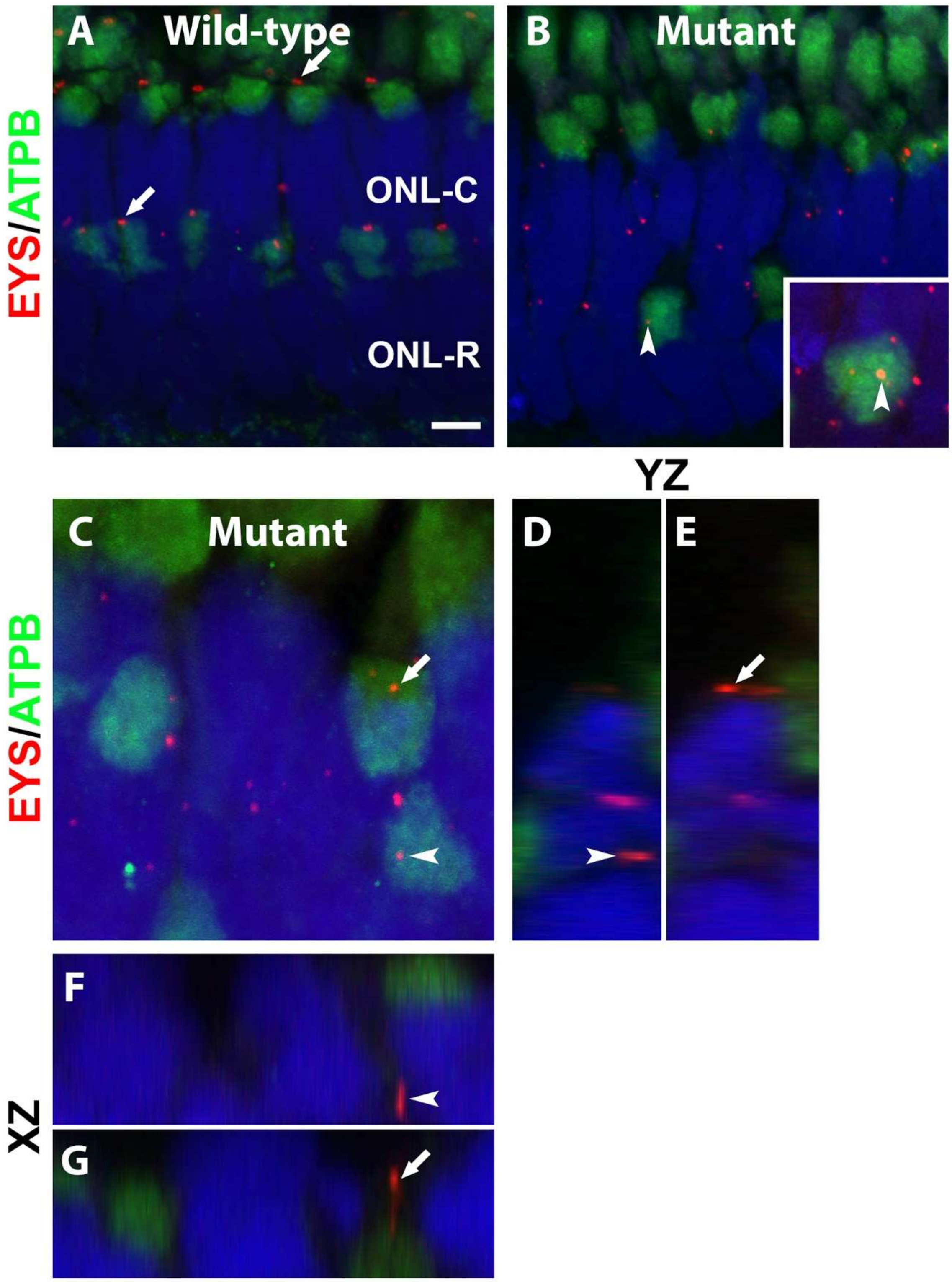

EYS/ATPB
A
Wild-type
ONL-C
ONL-R
B
Mutant
YZ
EYS/ATPB
C
Mutant
D
E
XZ
F
G

Supplementary Figure 2. Mislocalized EYS in *pomgnt1* mutant photoreceptors was not localized with late endosomes/lysosomes.

Cryosections of zebrafish eyes were double stained with anti-EYS (green) and with antibody against late endosome/lysosome marker VPS33B (red). The sections were counter-stained with DAPI to visualize nuclei (blue).

(A) Wild-type. In the wild-type retina, VPS33B antibody labeled the ellipsoid region of photoreceptors as well as apical portions of the outer plexiform layer. Most EYS immunoreactive puncta were observed on the apical side of VPS33B reactivity (arrows).

(B) Homozygous *pomgnt1*^{sny7} mutant. Most EYS immunoreactive puncta were localized in the outer nuclear layer. Some appeared to be over the VPS33B immunoreactive domain (arrowheads)

(C-H) Mutant. Max projection image and its orthogonal views of EYS puncta that appeared to be over the ATPB immunoreactive domains. Of 62 EYS immunoreactive puncta that appeared overlapping with VPS33B immunoreactivity, none were within the VPS33B immunoreactive domain, indicating that mislocalized EYS puncta were not located in late endosomes/lysosomes.

Scale bar in A: 4.36 μm for A-B, 2 μm for C.

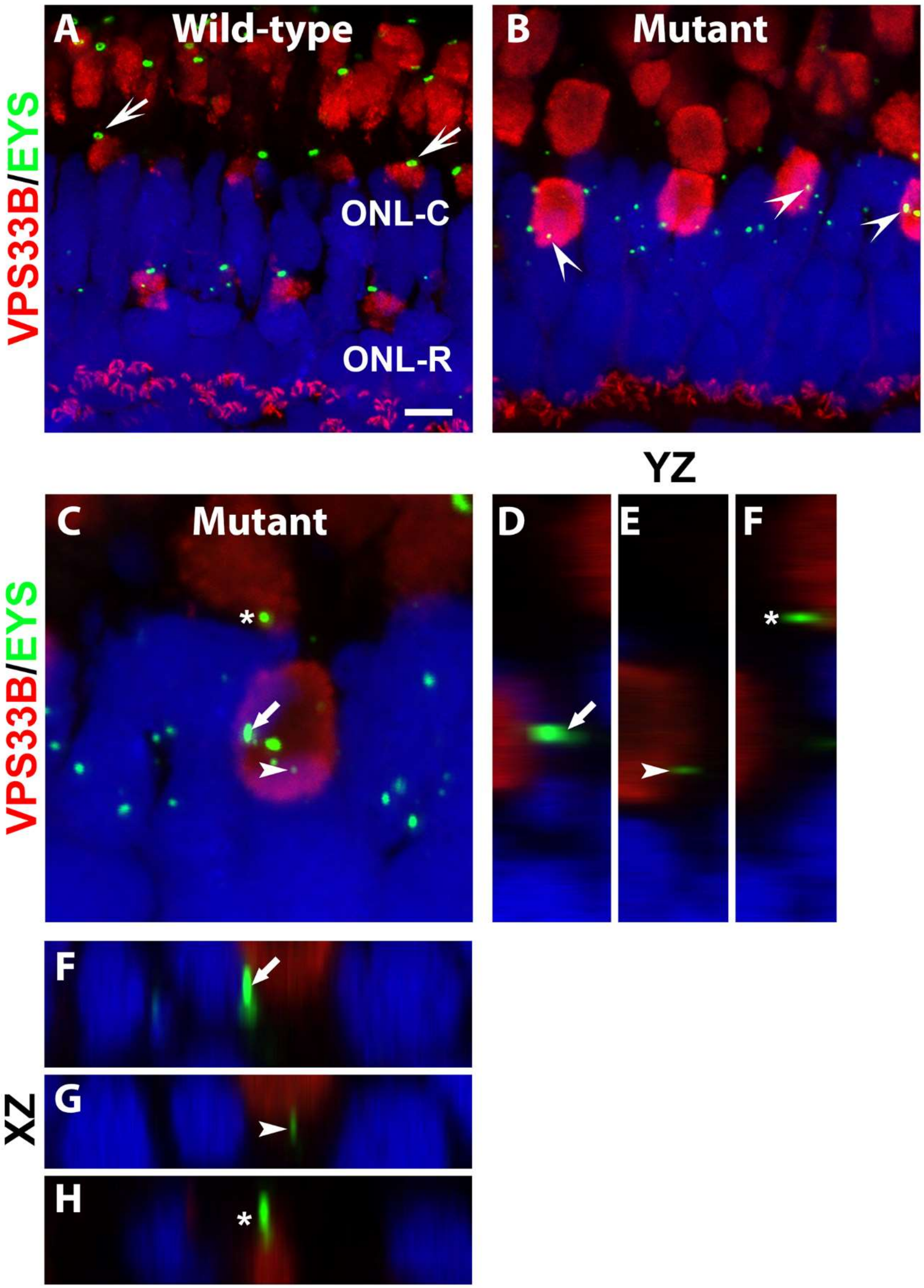
A
Wild-type
VPS33B/EYS
ONL-C
ONL-R
B
Mutant
YZ
C
Mutant
VPS33B/EYS
*
D
E
F
*
F
G
XZ
H
*

Supplementary Figure 3. Mislocalized EYS in *pomgnt1* mutant photoreceptors was not localized with the recycling endosomes.

Cryosections of zebrafish eyes were double stained with anti-EYS (red) and antibodies against recycling endosome marker Rab11a (green). The sections were counter-stained with DAPI to visualize nuclei (blue).

(A) Wild-type EYS and Rab11a double staining. In the wild-type retina, Rab11a immunoreactivity appeared as puncta. They did not overlap with EYS puncta.

(B) Homozygous $pomgnt1^{sny7}$ mutant EYS and Rab11a double staining. Most EYS immunoreactive puncta were localized to the outer nuclear layer. They did not overlap with Rab11a puncta, although a few EYS puncta appeared to overlap with Rab11a immunoreactive domain (arrow)

(C-I) Mutant. Max projection and its orthogonal views of EYS puncta that appeared to overlap with the Rab11a immunoreactive puncta. Of 20 EYS puncta that appeared to overlap with Rab11a immunoreactive puncta, none were co-localized with Rab11a reactivity.

Scale bar in A: 4.36 μm for A-B, 2 μm for C.

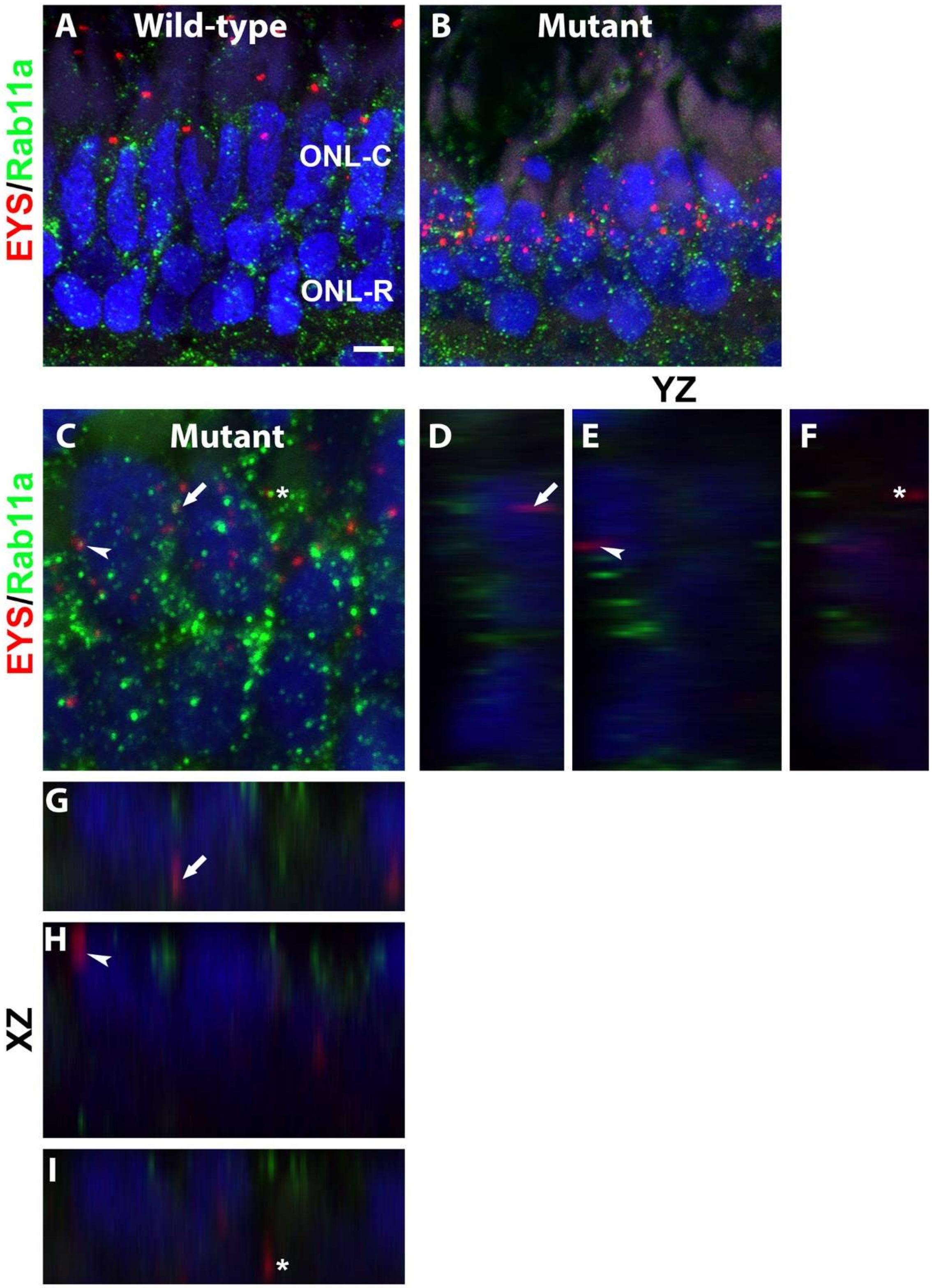
A Wild-type
B Mutant
EYS/Rab11a
ONL-C
ONL-R
YZ
C Mutant
D
E
F
EYS/Rab11a
G
H
XZ
I

Whole-length Western blot images of Figure 2 panels E, F, and G
(Green fluorescence:Blotting signal; Red fluorescence: Molecular weight marker in kDa)

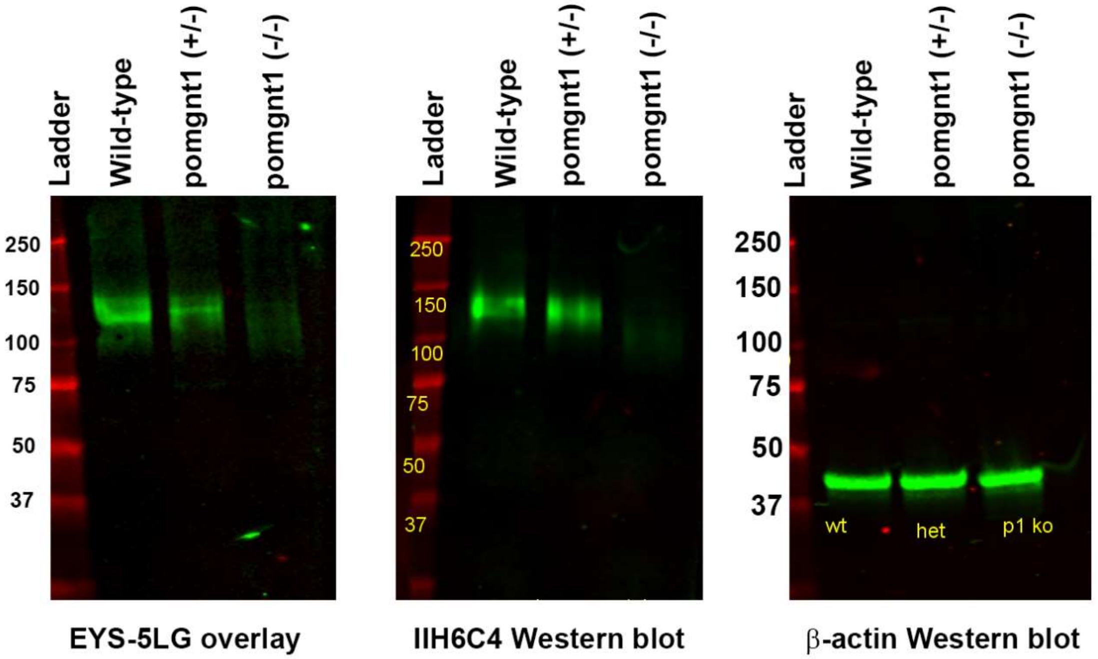

Supplement: Supplementary file 1 — Supplementary Information. [file 41598_2020_64752_MOESM1_ESM.pdf]
